# Endothelial Dysfunction and Liver Cirrhosis: Unraveling of a Complex Relationship

**DOI:** 10.3390/ijms252312859

**Published:** 2024-11-29

**Authors:** Antonio Nesci, Vittorio Ruggieri, Vittoria Manilla, Irene Spinelli, Luca Santoro, Angela Di Giorgio, Angelo Santoliquido, Francesca Romana Ponziani

**Affiliations:** 1 Angiology and Noninvasive Vascular Diagnostics Unit, Department of Cardiovascular Sciences, Fondazione Policlinico Universitario Agostino Gemelli IRCCS, 00168 Rome, Italy; 2 Liver Unit, CEMAD—Centro Malattie dell’Apparato Digerente, Medicina Interna e Gastroenterologia, Fondazione Policlinico Universitario Gemelli IRCCS, 00168 Rome, Italy; 3 Dipartimento di Medicina e Chirurgia Traslazionale, Università Cattolica del Sacro Cuore, 00168 Rome, Italy

**Keywords:** endothelial dysfunction, endothelium, cirrhosis, flow-mediated dilation, shear stress, albumin, statins, nitric oxide, Krüppel-like factor

## Abstract

Endothelial dysfunction (ED) is the in the background of multiple metabolic diseases and a key process in liver disease progression and cirrhosis decompensation. ED affects liver sinusoidal endothelial cells (LSECs) in response to different damaging agents, causing their progressive dedifferentiation, unavoidably associated with an increase in intrahepatic resistance that leads to portal hypertension and hyperdynamic circulation with increased cardiac output and low peripheral artery resistance. These changes are driven by a continuous interplay between different hepatic cell types, invariably leading to increased reactive oxygen species (ROS) formation, increased release of pro-inflammatory cytokines and chemokines, and reduced nitric oxide (NO) bioavailability, with a subsequent loss of proper vascular tone regulation and fibrosis development. ED evaluation is often accomplished by serum markers and the flow-mediated dilation (FMD) measurement of the brachial artery to assess its NO-dependent response to shear stress, which usually decreases in ED. In the context of liver cirrhosis, the ED assessment could help understand the complex hemodynamic changes occurring in the early and late stages of the disease. However, the instauration of a hyperdynamic state and the different NO bioavailability in intrahepatic and systemic circulation—often defined as the NO paradox—must be considered confounding factors during FMD analysis. The primary purpose of this review is to describe the main features of ED and highlight the key findings of the dynamic and intriguing relationship between ED and liver disease. We will also focus on the significance of FMD evaluation in this setting, pointing out its key role as a therapeutic target in the never-ending battle against liver cirrhosis progression.

## 1. Introduction

Cirrhosis is the endpoint of liver injury, which arises from multiple etiological factors (e.g., viral infections, metabolic overload, excessive alcohol intake, genetic and autoimmune disease) and is associated with several clinical complications. Endothelial dysfunction (ED), marked by impaired vasodilation and increased vascular stiffness, has been recognized as a pivotal component of cirrhosis pathophysiology. Indeed, the onset of cirrhosis is associated with profound vascular alterations that exacerbate disease progression and cardiovascular disturbances. At the beginning of this process, the gradual capillarization of hepatic endothelium leads to perisinusoidal fibrosis and portal hypertension. Then, a complex interplay between bacterial translocation, increased systemic circulation of pro-inflammatory molecules, and neurohumoral factors promotes peripheral vasodilation, increasing splanchnic accumulation of blood flow and a hyperdynamic systemic circulation characterized by decreased arterial blood pressure and expanded plasma volume. This circulatory dysfunction is initially compensated by an increased cardiac output, which later deteriorates in the advanced stages of cirrhosis because of cardiac insufficiency due to electrophysiologic and structural changes (i.e., cirrhotic cardiomyopathy) [1,2,3,4]. This review examines the endothelial pathophysiology, its role in the pathogenesis of liver cirrhosis, the mechanisms of ED development in this setting, its clinical consequences, and the potential of techniques used to assess endothelial function in cirrhotic patients, such as flow-mediated dilation (FMD).

## 2. Pathogenesis of Endothelial Dysfunction

The vascular endothelium is the innermost layer of the heart and blood vessels, and it acts simultaneously as a semi-permeable barrier and as a dynamic structure, crucial for regulating vascular health and homeostasis. Endothelial cells (ECs) have many physiological properties: they regulate vascular tone, hemostasis, inflammation, permeability, and oxidative stress, providing a natural barrier to bloodborne pathogens. So, under normal circumstances, the endothelium acts as an autocrine, paracrine, and endocrine organ, releasing messenger molecules to maintain an antioxidant, anti-inflammatory, anti-thrombotic, and anti-proliferative milieu [5,6].

**Figure 1:**
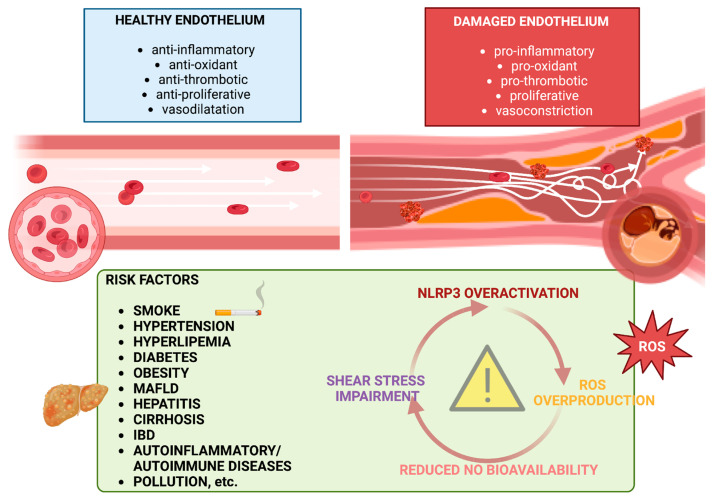
Main features of healthy and dysfunctional vascular endothelium. In homeostatic conditions, a healthy endothelium is a thin monolayer of endothelial cells at the interface between the circulatory system and tissues. Under normal circumstances, endothelium regulates vascular tone, hemostasis, inflammation, permeability, and oxidative stress, providing a natural barrier to bloodborne pathogens, thus maintaining an antioxidant, anti-inflammatory, anti-thrombotic, and anti-proliferative milieu. However, cardiovascular risk factors prompt the disruption of the endothelial barrier function through molecular and mechanic pathways such as inflammation, with the activation of NLRP3 and pro-inflammatory cytokine release, ROS overproduction and the consequent reduction in NO bioavailability, and shear stress impairment. This leads to deleterious vasoconstriction and a switch toward a pro-oxidant and proliferative condition, associated with increased vascular permeability, thrombosis, and inflammation. NLRP3: Nod-Like Receptor Protein 3; NO: nitric oxide; ROS: reactive oxygen species; IBD: inflammatory bowel diseases.

Historically, ED has been described as a polymorphic and multifactorial process, and the concept of morphological and functional gradual changes in endothelial function has been enriched by progressive acquisition in the literature. Hunt and Jurd recognized “five core changes of ECs activation” leading to ED: loss of vascular integrity; expression of leukocyte adhesion molecules; change in phenotype from antithrombotic to prothrombotic; cytokine production; and upregulation of human leukocyte antigen (HLA) molecules [7]. Since then, understanding invisible mechanisms behind ED has improved, and new pathways have been discovered, as described below and illustrated in Figure 1.

### 2.1. NO, ROS, and Inflammation

Many studies have recognized that a key role in ED is driven by a reduced bioavailability of vasodilator molecules, especially nitric oxide (NO). For several years, this molecule has been designated as an endothelial-derived relaxing factor (EDRF), and the discovery of its crucial role in vascular homeostasis and signaling earned Furchgott, Ignarro, and Murad the Nobel Prize in Physiology or Medicine in 1998 [8].

NO is a gaseous EDRF synthesized in ECs from L-arginine by three isoforms of the NO synthase (NOs), specifically by the endothelial isoform of NOs (eNOS). Vascular NO stimulates the soluble guanylyl cyclase that leads to increased cyclic guanosine monophosphate (cGMP) concentrations and the relaxation of smooth muscle cells; moreover, it produces the inhibition of platelet aggregation, leukocyte adhesion to the vessel wall, which is involved in atherogenesis, and smooth muscle cells proliferation. Another mechanism involved in ED is ROS overproduction, which consequently impairs NO synthesis. Under physiological conditions, ROS in ECs are produced by mitochondria, nicotinamide adenine dinucleotide phosphate (NADPH) oxidase (NOX), eNOS uncoupling, and xanthine oxidase, and they are essential for physiological cellular functions such as host defense, post-translational processing of proteins, cell signaling, regulation of gene expression, and cell differentiation [9,10].

Increased levels of ROS, and consequently weakened NO protective effect, are responsible for the production and secretion of pro-inflammatory cytokines, such as interleukin (IL)-1β, IL-18, IL-6, tumor necrosis factor (TNF)-α [11,12], and also induce adhesion molecules, such as vascular cell adhesion molecule 1 (VCAM-1), intercellular adhesion molecule 1 (ICAM-1), E-selectin, and monocyte chemoattractant protein-1 (MCP-1), thus enhancing leukocyte adherence and extravasation into the vessels wall [13,14].

In this way, the pro-inflammatory milieu created by oxidative stress promotes vasoconstriction and microvascular remodeling [15].

These vascular changes that promote inflammation and thrombosis are mainly driven by the activation of inflammasome Nod-Like Receptor Protein 3 (NLRP3) by reactive oxygen species (ROS). NLRP3 is a complex protein that detects various stimuli, including pathogens and their molecular products (PAMPs, pathogen-associated molecular patterns) and molecules released from damaged cells (DAMPs, damage-associated molecular patterns). There are two key mechanisms for NLRP3 inflammasome activation in the innate immune response: the canonical and noncanonical pathways. In the former, microbial elements and cytokines act as the first signal, activating the transcription factor NF-κB through toll-like receptor (TLR) ligands or cytokine receptors. This triggers the inflammasome by increasing the expression of NLRP3 itself and pro-IL-1β. This process is called the priming phase. Subsequently, the “activation phase” occurs when the NLRP3 inflammasome is stimulated by various factors, such as bacterial and fungal toxins, viral RNA, extracellular matrix components, adenosine triphosphate (ATP), potassium and chlorine efflux, calcium and sodium influx, lysosomal leakage, ROS, and mitochondrial dysfunction. At this point, NLRP3 oligomerizes with an ASC (apoptosis-associated speck-like protein containing a caspase recruitment domain, also known as CARD), which activates pro-caspase-1 to caspase-1, leading to the release of IL-18 and IL-1β. The noncanonical pathway, on the other hand, does not need TLR priming, but directly promotes the intracellular activation of caspase-11 in mice and caspases-4 and 5 in humans by LPS. Both pathways, canonical and noncanonical, lead to the caspase-mediated cleavage of gasdermin D, which triggers pyroptosis (a form of inflammatory cell death) and facilitates the release of IL-1β and IL-18 into the extracellular space [16,17].

ROS-NLRP3 cross-talk has been studied in cardiovascular conditions, such as hyperlipemia, hyperglycemia, smoke, and hypertension [18,19,20,21,22], but also in liver diseases, such as NAFLD [23,24,25], alcoholic liver disease (ALD) [26,27,28,29,30], associated with long-term alcohol abuse, and the hepatitis B (HBV) and C (HCV) viruses [31,32,33,34,35,36,37].

Thus, ROS has been demonstrated as a bridge between pathological stimuli, NLRP3 activation, inflammatory milieu, and programmed EC death, all leading to ED.

### 2.2. Flow Shear Stress

Another pillar of vascular health is flow shear stress (FSS), which refers to the mechanical forces in the vascular lumen and EC microenvironment.

The endothelium is permanently exposed to different mechanical forces: radial force, i.e., the hydrostatic pressure perpendicular to the vessel wall; tangential forces, i.e., the cell–cell adhesion and vessel vasomotion and axial shear force, i.e., the blood flow friction against the vessel wall. FSS can be expressed as the force per unit area determined by a tangential force (blood flow) on a surface (endothelium). From heart to capillaries, arteries are subjected to high and highly pulsatile FSS (up to 10–50 dyn/cm^2^), while veins are subjected to a more consistent flow (10-fold less). Several distinct flow patterns have been identified by altering flow characteristics, such as its amplitude and direction, along with changes in pump characteristics, such as velocity and pulse force. These include stable laminar flow, in which the fluid moves parallel along the major axis of the artery; disturbed laminar flow, which is characterized by flow splitting, recirculation, and reunification; and turbulent flow, which occurs when the motion is multidirectional and chaotic with continuous changes in velocity. [38,39]. Research indicates that high shear stress, which typically occurs in the rectilinear regions of arteries with undisturbed laminar blood flow, has a protective effect against atherogenesis: this stimulus promotes an anti-inflammatory environment, in which cells align with the direction of flow, express anti-inflammatory genes, provide antioxidant signals, are quiescent with reduced migration and proliferation, and show reduced permeability. In contrast, low or oscillatory shear stress, which is mainly found in geometrically irregular arterial regions (at curvatures, near branches, or bifurcations, upstream or downstream of stenoses) with laminar or turbulent flow, stimulates a pro-inflammatory and pro-thrombotic environment, characterized by poor cell alignment, the high production of cytokines and adhesion molecules, increased oxidative stress and NO impairment, and high cell turnover [5].

This consideration implies that FSS change sensing is fundamental to EC adaptation. Several mechanosensors have been studied, localized on the cell surface (primary cilia, glycocalyx, ion channels, G protein-coupled receptors (GPCRs), G-proteins, or protein kinases), at cell–cell junctions (platelet-endothelial-cell adhesion molecule (PECAM)-1 (CD31), vascular endothelial (VE)-cadherin, vascular endothelial growth factor receptor (VEGFR)-2 and -3), or at sites of cell–matrix adhesion [40].

As previously said, FSS regulates changes in vascular diameter. Pathological processes such as hyperlipidemia, hypertension, diabetes, and inflammatory disorders cause major disruptions of hemodynamic forces by pleiotropic effects, and this likely impacts mechanosensing pathways and their derived vascular phenotypes [41].

## 3. Endothelial Dysfunction in Liver Disease

### 3.1. Liver Sinusoidal Endothelial Cells: Key Players in Maintaining Intrahepatic Endothelial Function

**Figure 2:**
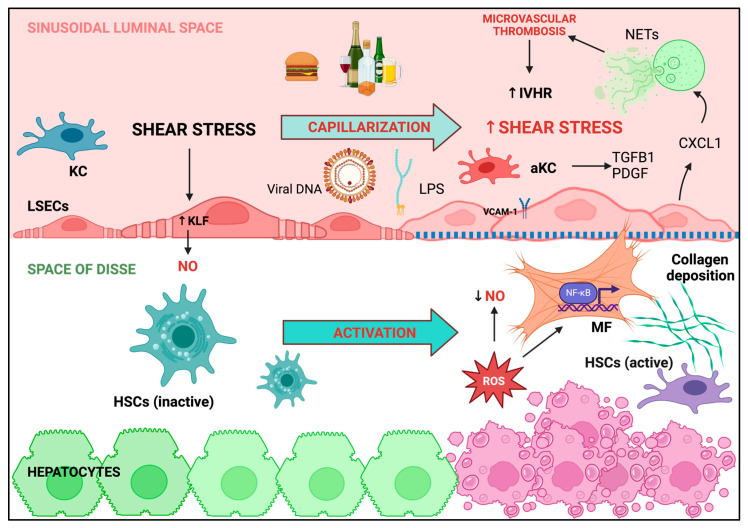
Intrahepatic endothelial dysfunction. In normal conditions, NO production by LSECs, physiologically driven by shear stress through the upregulation of KLF2 transcription, controls vascular tone, and mantains HSCs quiescence. In case of liver injury, aside from the etiology (i.e., alcohol, HFD, viral hepatitis, LPS), this delicate balance is disrupted; LSECs lose their fenestrae and acquire basal membrane in a process defined as “capillarization”. As a result, shear stress increases, but the intrahepatic concentration of NO decreases because of changes in LSEC phenotypes and aberrant ROS production by injured hepatocites. Within the sinusoidal lumen, KCs are activated by liver damaging agents, with the subsequent release of pro-inflammatory cytokines and chemokines and the increased expression of vascular adhesion molecules such as VCAM on LSECs, which further stimulate monocytes differentiation into inflammatory, angiogenic, and fibrogenic macrophages. Activated KCs promote collagen production by HSCs and MFs through the release of several mediators, such as TGFβ1 and PDGF, also capable of enhancing MF survival through the stimulation of NF-kB transcription, also triggered by the increased release of ROS. In parallel, the increase in shear stress interacts with mechanosensitive calcium channels promoting CXCL1 expression, a neutrophil chemoattractant; neutrophils promote NETs formation which favor microvascular thrombosis and liver injury progression, with subsequent IHVR increase and portal hypertension development. NO: nitric oxide; KLF: Krüppel-like factor; HSCs: hepatic stellate cells; ROS: reactive oxygen species; HFD: high fat diet; LPS: lipopolysaccharide; LSECs: liver sinusoidal endothelial cells; KC: Kupffer cell; VCAM: vascular cell adhesion molecule; TGF-β1: Transforming growth factor beta 1; PDGF: platelet-derived growth factor; NF-kB: nuclear factor kappa-light-chain-enhancer of activated B cells; CXCL1: chemokine (C-X-C motif) ligand 1; MF: myofibroblasts; IHVR: intrahepatic vascular resistance.

ED is not only responsible for the typical manifestations of cardiovascular disease, but it also plays a pivotal role in the development of liver disease and its complications (Figure 2).

The highly specialized liver sinusoidal endothelial cells (LSECs) that represent the most part of portal intrahepatic endothelium are the main protagonist [42]; placed at the end of the portal venous system, LSECs collect and filter most of the substances deriving from the gut, representing the most effective scavenger ECs of the human body [43]. The constitutional lack of the basal lamina, the presence of multiple fenestrae, and the complex endocytic system allow the passage of molecules between the sinusoidal lumen and the underlying hepatocytes [44], making LSECs crucial in multiple metabolic pathways, as well as in modulating immune cell functions and the inflammatory response [45]. LSECs also constitute the main source of hepatic NO [46]; as mentioned above, this small molecule is produced in response to shear stress and is capable of regulating sinusoidal vasodilation through cyclic cGMP formation; within the liver, it also maintains the quiescence of hepatic stellate cells (HSCs) and promotes hepatocytes fatty acid β-oxidation [47], a vasoprotective metabolic pathway [48]. LSECs are exposed to potentially harmful substances that can disrupt their delicate endothelial function throughout the activation of multiple inflammatory pathways, as discussed below. Inflammation drives chronic liver damage and is always associated with progressive LSEC dedifferentiation, with the loss of fenestrae and the acquisition of a basement membrane, a phenomenon called LSEC capillarization, invariably associated with HSC activation [49]. This organ-specific form of ED is responsible for the increased intrahepatic resistance to portal blood flow, leading to portal hypertension. Indeed, in recent years, it has become clear that portal hypertension not only originates from intrahepatic structural changes, leading to fibrosis and parenchymal nodularization (the mechanical component), but also relies on LSEC dysfunction, which sustains the dynamic and reversible component of intrahepatic resistance, thanks to a continuous interplay between sinusoidal and extra-sinusoidal contractile elements, but also to activated HSCs and hepatic myofibroblasts [50], both protagonists of the hepatic inflammatory response that leads to liver fibrosis [51,52].

### 3.2. Hepatic Macrophages Trigger the Inflammatory Pathways Involved in LSECs Dysfunction and Liver Disease Progression

Hepatic macrophages, such as Kupffer cells (KCs) and monocyte-derived macrophages, are considered the first-line defense against liver damaging agents, responsible for the initiation of the inflammatory responses that eventually lead to fibrosis [53,54]. In case of chronic liver injury, KCs are activated by various damage-associated DAMPs (e.g., free DNA, free fatty acids, hypoxia) and PAMPs (e.g., LPS or viral DNA), and they assemble NLRP3 inflammasome, responsible for releasing multiple pro-inflammatory cytokines and chemokines, such as IL-1β and IL-18 [55,56]; this results in the recruitment of circulating leukocytes, the modulation of T cell response, and the increased expression of vascular adhesion molecules such as I-CAM or V-CAM on LSECs, which can also induce the differentiation of monocytes into inflammatory, angiogenic, and fibrogenic macrophages. On their hand, activated macrophages promote collagen production by HSCs and myofibroblasts through the release of several mediators, such as transforming growth factor (TGFβ1) and platelet-derived growth factor (PDGF), also enhancing myofibroblasts survival through the stimulation of nuclear factor kappa B (NF-kB) transcription. The same pathway is triggered by the aberrant release of ROS from injured hepatocytes, with the contribution of superoxide dismutase (SOD) decreased scavenging action [57]. The role of NOX regulation in diminishing NO concentration, despite its leading role in ROS production, has been questioned; indeed, a decreased expression of NADPH oxidase 4 (NOX4), one of the main isoforms expressed in ECs but not clearly in KCs or macrophages [58], has been found in the intrahepatic vascular system of cirrhotic livers. If NOX does not seem to play a direct role in modulating the intrahepatic vascular tone in cirrhosis, as its inhibition mediated by apocynin cannot reduce liver fibrosis [59], many studies point out NOX isoform upregulation as responsible for ROS increased production leading to hepatic fibrosis [60,61,62], and someone went even further, demonstrating that Angiotensin II—another key player in ED development—may be responsible for NOX upregulation, leading to HSC activation and ROS increased levels [63,64].

### 3.3. ROS Overproduction Favors Intrahepatic Thrombosis

ROS overproduction and subsequent diminished NO levels are responsible for a substantial decrease in the inhibition of thrombogenesis by dysfunctional LSECs; the attenuated expression of thrombomodulin, NO, and prostaglandin I2 is associated with the increased exposition of von Willebrand factor (vWF), integrins, and other receptors that cause clot formation by interacting with activated platelets [65,66], suggesting that liver fibrosis may result from microvascular thrombosis. More specifically, it has been proposed that the disruption of blood flow with sinusoidal microthrombi causes reactive hyperemia and congestion, which activate fibroblasts and increase collagen deposition, worsening blood flow, inducing the extension of thrombosis, neoangiogenesis, and hepatocyte necrosis, leading to the so-called “parenchymal extinction” [67]. Exploring the critical role of the pro-thrombogenic milieu that accompanies the progression of liver disease and the development of portal hypertension is essential when searching for new, noninvasive, and reliable tools to detect CSPH. In a pediatric population of patients with CSPH, Goel et al. found that vWF can be used to predict clinically significant varices, variceal bleeding, and disease compensation in association with vWF glycoprotein Ib binding activity (GPIbR) and transient elastography [68]. Similar data about the predictive value of vWF increase in pediatric CSPH were also reported [69]. As for the adults, a 2019 meta-analysis compared HVPG and vWF accuracy in detecting CSPH and found a moderate correlation between them, suggesting vWF satisfactory performance for the diagnosis of CSPH in cirrhotic patients [70].

### 3.4. Shear Stress and Neutrophils Regulate of LSECs Function

Shear stress, whose magnitude depends on the blood flow and the area of the intrahepatic sinusoids [71], physiologically drives Kruppel-like factor 2 (KLF2) transcription, which is responsible for NO production and is markedly increased in the liver of cirrhotic rats as negative feedback to counteract the vascular derangement occurring in advanced stage liver disease [72]. Liver fibrosis develops faster when KLF2 transcription cannot be increased [73]. As further proof of its fundamental role in maintaining LSECs function, Guixé-Muntet et al. demonstrated that, in case of liver ischemia due to cold preservation or surgical procedures, there is a rapid loss of KLF2 transcription accompanied by LSEC capillarization and microvascular alteration, overall leading to liver injury; the use of KLF2-inducing agents (statins, resveratrol, shear stress) can help to prevent this negative effect [74]. Another confirmation of how similar LSECs dysfunction is to systemic ED, a decrease in KLF2 has been recently implicated in the pathogenesis of heart hypertrophy and cardiac failure through neutrophil activation [75]. Notably, neutrophils appear to be involved in congestive liver disease progression; the increase in shear stress can interact with mechanosensitive calcium channels that increase the expression of chemokine (C-X-C motif) ligand 1 (CXCL1), a neutrophil chemoattractant, which promotes the formation of neutrophil extracellular traps (NETs) involved in microvascular thrombosis and the progression of liver injury and portal hypertension [76]. NASH progression has been linked to parenchymal neutrophil infiltration and NET development in mice, too [77], and it has been widely recognized that LPS can induce NET formation [78] and maintain the vicious circle in which portal hypertension increases intestinal permeability that promotes bacterial and LPS translocation, worsening inflammation and liver decompensation [79]. Not surprisingly, increased LPS levels have also been associated with low-grade inflammation linked to principal metabolic diseases marked by ED [80]. As for the liver, it is widely recognized that a leaky gut is involved in the worsening of liver function in cirrhosis [81]. Recently, using eight different models of liver damage showed that the disruption of the intestinal vascular barrier (GVB) in models of sodium dextran sulfate (DSS)-induced colitis has no effect on liver damage when the function of LSECs is preserved. In contrast, when LSECs are disrupted, liver damage is significantly exacerbated by colitis and inevitably associated with the increased expression of CXCL1 and the subsequent accumulation of hepatic neutrophils, both of which are stimulated by the increased expression of LPS (or endotoxin) and the overproduction of TNF-alpha [82]. As mentioned above, activated neutrophils give rise to NETs and promote thrombosis, while all leukocytes, attracted by increased LPS levels, attach to the sinusoidal lining and impair the perfusion of LSECs, resulting in their vasodilator hyporesponsiveness and vasoconstrictor hyperresponsiveness [83].

### 3.5. Bile Acids: New Key Players in Endothelial Dysfunction Occurrence

**Figure 3:**
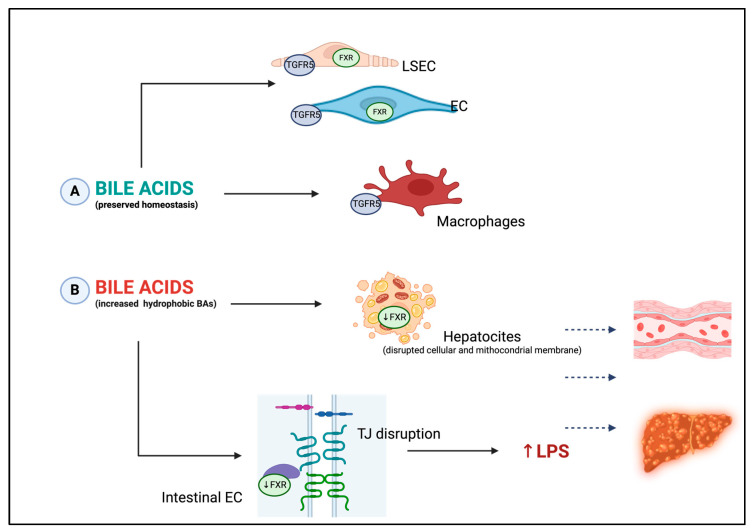
(**A**). Bile acid (BA) homeostasis is essential to preserve not only the liver homeostasis, but also the endothelial function. Among BA receptors, TGR5 and FXR are the most studied. TGR5 is expressed not only on hepatocytes cellular membrane, but also on LSECs endothelial cells and macrophages and exert their vasodilatory action by increasing NO expression and decreasing ET-1 levels; furthermore, it preserves the hepatic and endothelial function by modulating cytokine secretion. (**B**). When BA homeostasis is disrupted and their composition changes, altering the balance between the hydrophobic and the hydrophilic component, cellular and mitochondrial membranes of hepatocytes and intestinal cells can be damaged, causing apoptosis. In the gut, BAs alter tight junctions and increase intestinal permeability, bacterial translocation, and lipopolysaccharide concentration. Together with the direct damage of the liver, the activation of these pathways is responsible for the increased intrahepatic vascular resistance that accompanies liver disease progression and the endothelial dysfunction. BAs: bile acids; NO: nitric oxide; LPS: lipopolysaccharide; ECs: endothelial cells; LSECs: liver sinusoidal endothelial cells; TGR5: Takeda G-protein associated receptor TGR5; FXR: Farnesoid X receptor; ET-1: Endothelin-1; TJ: tight junction.

Bile acids (BAs) are amphipathic cholesterol metabolites that are synthesized in the liver, stored in the gallbladder, and released after feeding into the small intestine, where they regulate lipids and lipid-soluble vitamins absorption [84]. A diet rich in fats results in higher levels of liver cholesterol and increased BAs synthesis. The consequences of altered BAs metabolism involve (a) the accumulation and impaired detoxification of secondary hydrophobic BAs, which decreases the expression of tight junctions (TJ) increasing intestinal permeability, with subsequent translocation of endotoxin to the portal circulation; (b) hepatocyte and enterocyte apoptosis due to the interaction between hydrophobic secondary BAs and extracellular and mitochondrial membrane phospholipids [85]. In recent years, BAs were also proven to be vasoactive compounds with vasodilatory effect, which has been confirmed by multiple in vitro studies; this is associated with different endothelial pathways that modulate NO production and inhibit endothelin-1 (ET-1) release [86] and extends to systemic circulation as part of the gut–liver–heart axis derangement [87,88]. BAs exert many of their functions through specific receptors, such as the nuclear farnesoid X receptor (FXR) and Takeda G-protein associated receptor TGR5, as described in Figure 3 [86]. TGR5 has been found to be expressed in macrophages (including KCs) and in LSECs, suggesting a direct action of BAs on LSECs through eNOS modulation and ET-1 inhibition again [89]; through TGR5, BAs are thus capable of protecting the liver through the control of their hydrophobicity and cytokine secretion, as elegantly resumed by Keitel et al. [90]. Its influence appears to not be limited to the liver, as multiple studies showed its protective role in multiple diseases associated with ED (i.e., diabetes, obesity, and cancer). Indeed, TGR5 acts on different molecular pathways and modulates multiple cell types, such as endothelial cells, cardiomyocytes, and smooth muscle cells [91]. As for nuclear FXR, TGR5 is strongly activated by conjugated BAs [92], and it can be found within the liver, inside the intestinal epithelial cells and the endothelial cells. Its expression, in association with bile acids, modulates NLRP3 inflammasome activation, as seen in cholestasis-induced sepsis [93], and it also induces eNOS expression, the formation of NO, and the decrease in ET-1 expression acting on the endothelium, suggesting that BAs may produce vasodilation via FXR too [94]. Furthermore, FXR activation on rat aortic smooth muscle cells also leads to the increased expression of the angiotensin II type 2 receptor, with its vasodilatory function [95], with the possible beneficial effect of FXR agonists on atherosclerosis [96], diabetes, and metabolic syndrome [97], even if literature on this topic is still poor.

### 3.6. Nitric Oxide in the Pathogenesis of Portal Hypertension

Portal hypertension onset in liver cirrhosis is defined by portal venous pressure values > 5 mmHg that progressively lead to the development of splanchnic arterial vasodilation; as previously explained, this triggers systemic cardio-vascular changes that ultimately foster the so-called “hyperdynamic state”, characterized by high cardiac output and low peripheral resistance. In this context, the overload of blood volume that reaches the liver encounters high intrahepatic resistance and further increases portal pressure, with a risk of developing clinically significant portal hypertension (CSPH) [50]. The conventional view of portal hypertension pathophysiology has also been described using the “NO paradox”, referring to the reduced NO availability inside the liver with subsequent intrahepatic vasoconstriction and elevated NO production in the peripheral systemic circulation, promoting arterial vasodilation and new vessel formation. Recent studies highlight that the entire NO/cGMP pathway plays a crucial role in this condition and suggest the use of cGMP serum/plasma levels as non-invasive markers of CSPH. Enrolling a total of 110 participants, including 70 patients with cirrhosis and portal hypertension, Sturm et al. measured cGMP levels and found a significant increase in cirrhotic patients with CSPH compared with cirrhotic patients without CSPH; notably, the increase was also significant in the subgroup of cirrhotic patients with esophageal varices detected at screening endoscopy who had no previous manifestations of portal hypertension [98], supporting the idea of using serum cGMP levels as a noninvasive biomarker of portal hypertension, especially to estimate the risk of esophageal varices. Further studies are needed to understand whether the targeting of this molecule can be used in new therapeutic approaches, e.g., phosphodiesterase type 5 (PDE5i) inhibitors and sGC stimulators, acting on the dynamic component of portal hypertension to reduce portal pressure and reverse liver fibrosis [99]. Notably, the same pathway has been related to KC activation and insulin resistance [100], and it was suggested as a possible therapeutic target in cardiovascular ED-related diseases [101,102,103,104].

## 4. Assessment of Endothelial Dysfunction: The Role of Flow-Mediated Dilation

Several methods of assessing endothelial and microvascular function (and dysfunction) in human studies have been used in recent decades.

Currently, the FMD assessment of the forearm is the most widely used non-invasive method. It consists of the ultrasonographic evaluation of brachial artery dilatation by measuring the diameter at rest and after reactive hyperemia, either after 5 min arterial occlusion with a distal inflated cuff distal around the forearm to the supra-systolic level (endothelium-dependent vasodilation) or after the administration of a NO-donor, such as nitroglycerin (endothelium-independent vasodilation). Thus, FMD expresses the percentage change from the baseline diameter mediated by NO in response to the increase in shear stress. Despite the simplicity of this procedure, high intra- and inter-observer variability, which requires well-trained, experienced operators and strict protocols, and factors influencing the reliability of the measure, such as medication, physical, and environmental effects, can challenge its practical application [105]. Mainly due to the “NO paradox” and hyperdynamic circulation, the use of FMD for the evaluation of ED in liver cirrhosis has led to controversial data [106]. Multiple studies found an increased FMD in cirrhotic patients, apparently ameliorating with liver disease worsening [107,108]; on the contrary, other studies observed a decreased FMD in cirrhotic patients, with an improvement after rifaximin treatment in patients affected by minimal hepatic encephalopathy, implying a role of the gut microbiota in the promotion and perpetuation of the inflammatory condition associated with the progressive worsening of both ED and CSPH in cirrhotic patients [109,110]. Only some of these studies distinguished patients based on the etiology of liver disease, and they all declared no significant differences in FMD except for non-alcoholic steatohepatitis (NASH) (Berzigotti et al. 2013 [111]), in which FMD was decreased when compared to other causes of liver disease. Gbaruko et al. analyzed FMD differences in patients with alcoholic steatohepatitis (ASH) with and without portal hypertension, finding a decreased FMD in the second population [112]. Regarding non-alcoholic fatty liver disease (NAFLD), its new definition as a metabolic dysfunction-associated steatotic liver disease (MASLD) [113] clearly defines its relationship with metabolic disorders, which are typically linked to systemic inflammation and increased cardiovascular risk [114]; FMD data are not in contrast with this indissoluble association, as demonstrated in a recent meta-analysis [113,115]. These findings lead us to wonder if liver disease progression is responsible for ED worsening or, on the contrary, if a more severe ED may promote liver disease progression; the missing piece of this complex puzzle could be found in intrahepatic arterial endothelial function, which is poorly studied despite its critical role in preventing intrahepatic hypoxia that triggers LSEC dysfunction and fibrosis progression, as demonstrated during ischemia/reperfusion injury and perilesional fibrosis progression following transarterial chemoembolization (TACE) for the treatment of hepatocellular carcinoma [116,117].

## 5. New Therapeutic Approaches in Liver Cirrhosis: An Emerging Relationship with Improved Endothelial Function

**Figure 4:**
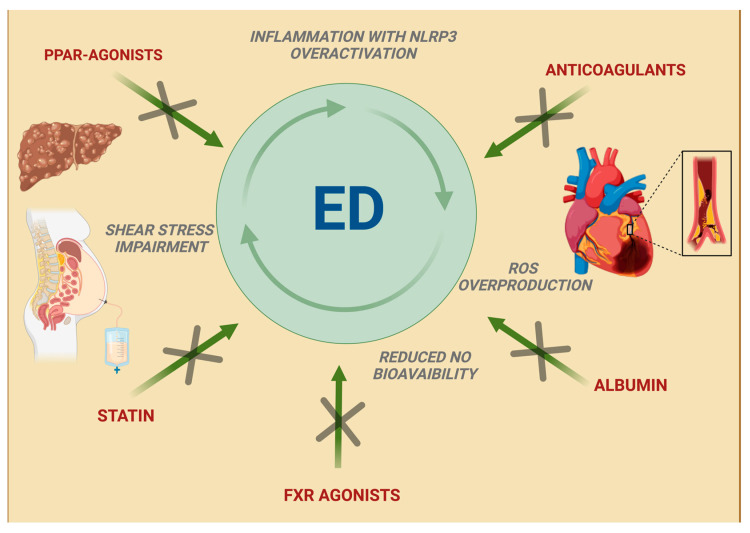
Experimental therapies used against liver cirrhosis and clinically significant portal hypertension have proved benefits in relieving endothelial dysfunction and its vicious circle, made up of inflammation, pro- and antioxidant imbalance, and shear stress impairment. Albumin has shown to exert non-oncotic properties with antioxidant, anti-inflammatory, and immunomodulatory features. Statins have demonstrated a role as anti-fibrotic, anti-proliferative, and antioxidant agents, thus ameliorating LSEC function and decreasing intra-hepatic resistance. Anticoagulants and PPAR agonists, such as pioglitazone and fibrates, have shown to improve portal hypertension through anti-angiogenetic, anti-inflammatory and anti-proliferative pathways. FXR agonists remodulate bile acid homeostasis with the subsequent preservation of hepatocytes and amelioration of the endothelial and gut–vascular barrier function. LSECs: liver sinusoidal endothelial cells. PPAR: peroxisome proliferator-activated receptor.

Although conflicting results exist in evaluating ED in liver cirrhosis through FMD measurements, classic and experimental therapies against CSPH and liver disease embrace the theory that liver cirrhosis and portal hypertension-related complications can be relieved by improving endothelial function (Figure 4). Specific medications, such as albumin, are known to exert an effect on endothelial function, but beneficial results have also been obtained with cardiovascular disease (CVD) risk-lowering therapies, which gained interest as additional treatments for liver cirrhosis.

### 5.1. Albumin

Albumin, mostly prescribed after large-volume paracentesis, in refractory ascites or in case of hepatorenal syndrome, exerts its non-oncotic properties through its antioxidant and scavenging functions, binding and carrying exogenous and endogenous substances [118] with subsequent improvement of ED and inflammatory response, as confirmed in the ANSWER study [119]. According to the PRECIOSA study, long-term treatment with high doses (1.5 g/kg every week for 12 weeks), but not with low doses, of albumin has been shown to increase left ventricular function and reduce IL-6 serum levels, thus lowering inflammation without changing portal pressure; the INFECIR-2 study, which compared antibiotic treatment plus short-term albumin administration with antibiotics alone in patients with bacterial infections (except for bacterial spontaneous peritonitis), confirmed the beneficial effect of albumin on cardiovascular function associated with decreased levels of inflammatory cytokines [120]. In the HEAL study, Bajaj et al. evaluated albumin’s beneficial effects on hepatic encephalopathy, showing its positive impact on patients’ quality of life; these results were associated with the reduction in IL-1β serum levels in the albumin group, as well as with an improved endothelial function, demonstrated by ED serum markers (ADAM, ICAM-1) reduction [121].

### 5.2. Statins

Statins, traditionally regarded as possible hepatotoxic agents to be used with caution in patients with chronic liver disease [122], are now considered probable antifibrotic agents that can reduce the risk of cirrhosis decompensation. In the liver of cirrhotic rats, simvastatin administration leads to KLF2 upregulation with the subsequent improvement in HSCs phenotype, in particular reducing the expression of α-smooth muscle actin and procollagen I and improving oxidative stress; in addition to reducing the fibrogenic potential, these changes ameliorate LSECs function, thus decreasing intrahepatic resistance, probably through a VEGF-mediated mechanism [123]; furthermore, in the specific context of LPS-induced sepsis, the administration of simvastatin showed better LSEC and peripheral endothelial function in rats, with the increase in thrombomodulin levels translating into a significant antithrombotic effect on liver microcirculation and the amelioration of peripheral hypo-coagulability [124]. In cirrhotic patients, one-month treatment with simvastatin decreases HVPG, improves liver perfusion without significant adverse events, and provides an additional effect to beta-adrenergic blockers [125]. This suggests the need for studies on long-term statin administration in cirrhotic patients; to make this possible, the LIVERHOPE-SAFETY phase 2 trial analyzed simvastatin adverse events in liver cirrhosis. The use of high doses of simvastatin (40 mg/die) was discouraged, even if there was no reported difference in terms of safety between the group treated with simvastatin 20 mg and the placebo group [126]. Simvastatin 20 mg plus rifaximin treatment was also proven to decrease protein catabolism and reduce lipids and fatty acid plasma levels, possibly reflecting an improvement in mitochondrial function, with a concomitant decrease in plasma concentrations of secondary bile acids (BAs) [127,128]. Based on these findings, in the ongoing SACRED phase III trial, patients with compensated cirrhosis and CSPH, stratified on the concomitant use of nonselective beta-blockers, are randomized to simvastatin 40 mg/day versus placebo for up to 24 months; during this period, the development of hepatic decompensation (i.e., variceal hemorrhage, ascites, encephalopathy), hepatocellular carcinoma, liver-related death, death from any cause, and complications of statin therapy will be recorded [129]; these and other studies will clarify the usefulness of prescribing statins in cirrhotic patients, ahead of their CVD risk.

### 5.3. Peroxisome Proliferator-Activated Receptor Agonists

Among peroxisome proliferator-activated receptor (PPAR) agonists, pioglitazone showed a positive effect in two rat models of cirrhotic and non-cirrhotic portal hypertension. In particular, the observed reduction in portosystemic shunting after treatment was associated with the inhibition of splanchnic neoangiogenesis and the downregulation of pro-inflammatory pathways together with the development of a more mature vasculature; the avoided increase in portosystemic collateral blood flow in fact prevents toxins, bacterial products, and drug shunting, which worsens inflammation and portal hypertension. Furthermore, pioglitazone inhibits portosystemic collateral formation without an increase in portal pressure due to beneficial effects on sinusoidal remodeling [130]. Not surprisingly, in a recent meta-analysis, pioglitazone alone or in association with vitamin E showed a very high probability of being ranked the most effective intervention for MASH resolution without worsening fibrosis [131]. Similar encouraging results were obtained in cirrhotic rats with fenofibrate, an agonist of PPARα, which regulates genes related to vascular tone and oxidative stress. Fenofibrate attenuates septal thickness and increases nodule size, while a wider septal thickness and lower nodule size have been associated with a more severe degree of portal hypertension, with a concomitant reduction in HSC activation [132]; in a study by Ogata et al., fenofibrate also showed its beneficial action on intrahepatic ED by reducing COX-1 expression and subsequent thromboxane A2 (TXA2) production, and it was associated with a significant increase in NO bioavailability and increased eNOS activation in ECs and cGMP levels in the analyzed tissue [133].

### 5.4. Anticoagulants

The use of anticoagulants in patients with liver cirrhosis is often debated due to the belief that a low platelet count and portal hypertension can increase the bleeding [134,135,136,137,138,139]. Moreover, prolonged prothrombin time (PT) and activated partial thromboplastin time (aPTT) observed in cirrhosis have led for many years to the misconception of a natural condition of impaired coagulation in these patients. However, in a 12-month randomized controlled trial, enoxaparin demonstrated its safety in cirrhotic patients with a Child–Turcotte–Pugh (CTP) score of 7–10, showing also the capacity to delay the occurrence of hepatic decompensation and to improve survival [140]. Heparin interferes in the fibrogenic pathway driven by inflammation and thrombosis, which acts on HSCs and myofibroblasts [141], but it also blocks VEGF proliferative effects in the presence of VEGF-induced NO production, thus stabilizing the endothelial function without stimulating vessel proliferation [142]. The activation of protease-activated receptors (PARs), upregulated in case of liver injury, on HSCs and myofibroblasts leads to the overproduction of extracellular matrix components. Nowadays, it is recognized that either thrombin or factor Xa are PARs agonists, while PARs inhibition results in a significant amelioration in HSCs phenotype and hepatic fibrosis progression [143]. Through its action on thrombin, the prolonged administration of enoxaparin may promote fibrosis regression and improve CSPH, as demonstrated in animal models [144], although this was not confirmed by Fortea et al., who reported no effects on liver fibrosis and hemodynamics, nor influence on systemic inflammation and bacterial translocation, and observed deleterious effects of enoxaparin treatment. Analyzing their results and those coming from previous studies, the authors suggested enoxaparin pharmacokinetics and/or pharmacodynamics alterations in advanced liver disease as a possible cause of their findings, postulating the presence of a limited therapeutic window to be considered in clinical and experimental studies [145]. In this regard, the cirrhotic population shows a reduction in antithrombin levels directly related to the degree of liver dysfunction and reasonably responsible for the ineffectiveness of heparin, as demonstrated in other situations [146]. In contrast to low-molecular-weight heparin (LMWH) such as enoxaparin, rivaroxaban is an antithrombin-independent inhibitor with highly selective activity against Factor Xa, thus with antithrombin-independent effects; 2 weeks of administration of rivaroxaban was able to decrease portal hypertension in two preclinical models of cirrhosis, and this effect is mainly achieved by improving intrahepatic vascular resistance by increased NO bioavailability, HSCs deactivation and decreased liver micro-thrombosis, leading to the amelioration of LSECs function and the reduction in portal pressure without negative effects on systemic hemodynamics or bleeding [147]. Regarding direct oral anticoagulants (DOACs), in a recent comprehensive review, all direct FXa inhibitors appear to have an acceptable safety profile for patients with CTP A cirrhosis, while apixaban and edoxaban seem to be more appropriate for patients with CTP B cirrhosis. The data available on dabigatran are insufficient to offer any recommendation. As for rivaroxaban, its efficacy and safety in CTP B patients needs to be further investigated because in vitro data demonstrate increased levels in these populations [148]; however, preliminary results of the CIRROXABAN trial, which evaluates rivaroxaban capacity to prevent thrombotic events in patients with liver disease and portal hypertension, suggest that a prophylactic dose of rivaroxaban may be safe.

### 5.5. Farnesoid X Receptor Agonists

Literature is rich with animal models studies and review articles demonstrating that FXR agonists can improve liver fibrosis by disrupting multiple pathways that lead to intestinal permeability and chronic inflammation [149,150], making this drug class a promising weapon against MASLD and cholestatic diseases that find pruritus the most common adversarial effect [151,152,153,154,155]. However, despite the evident FXR action on vascular tone modulation and ED, data regarding its effectiveness and safety in advanced liver disease and portal hypertension are still missing. Studies in cirrhotic rats showed a decrease in both BA levels and portal pressure in animals treated with FXR agonists; although there was no identified specific molecular target, they confirmed the ability of FXR agonists to preserve the gut–vascular barrier and modulate the endothelial function by ET-1 inhibition and NOS expression regulation [156]. In another animal model analyzing the effect of the FXR agonist cilofexor on NASH fibrotic rats, a decrease in portal pressure without changes in splanchnic blood flow or systemic hemodynamics occurred after the addition of propranolol; while cilofexor seems to primarily decrease sinusoidal resistance in cirrhotic portal hypertension, the combination of the two drugs reduced splanchnic inflow, but also the mean arterial pressure and heart rate [157].

## 6. Discussion

ED is not only the undisputed playmaker of the most common metabolic and cardiovascular diseases, but also in the onset and progression of liver fibrosis and portal hypertension, aside from specific liver disease etiology [42].

LSEC dysfunction is sustained by a continuous and intricate interplay between different cell types and molecules; hepatocytes, HSCs, myofibroblasts, neutrophils, KCs, and other activated leukocytes, but also BAs and endotoxin acting on the other side of GVB all play a pivotal role in favoring fibrosis progression through the constitution of a pro-inflammatory and thrombogenic milieu, invariably characterized by the increase in ROS production and shear stress that leads to increased IVHR up to portal hypertension occurrence. When it develops, the “NO paradox” is peculiar to the complex molecular milieu of cirrhotic patients. In fact, on the one hand, the decrease in hepatic NO bioavailability with the increase in intrahepatic resistance is an ascertained mechanism involved in the onset of portal hypertension and in the establishment of a systemic hyperdynamic circulation [42,50]; on the other hand, the paradoxical increase in NO concentration in peripheral arteries is the main confounding factor in FMD assessment in cirrhotic patients, since it primarily relies on NO-dependent vasodilation of peripheral arteries in response to “shear stress”. Thus, evaluating the entire NO/cGMP pathway could be a valuable option to overcome this notorious paradox and obtain a more accurate picture of circulatory changes occurring during liver disease progression, as suggested by the experimental use of cGMP serum/plasma levels as a non-invasive marker of CSPH [99].

Nevertheless, despite the complexity of these molecular and hemodynamic modifications, FMD evaluation in liver cirrhosis must be encouraged as an easy-to-use and non-invasive marker of ED. The undeniable resemblance between the molecular pathways in CVD and liver cirrhosis underlines the need to study this ambivalent and intricate relationship further. In fact, FMD could also serve as a non-invasive tool for assessing subclinical cardiovascular risk in cirrhotic patients, improving outcomes before and after liver transplantation [158]. Moreover, a systematic ED evaluation in early and advanced stages of liver disease could improve the knowledge of its pathogenetic pathways and support the role of the above-mentioned therapeutic agents, all capable of counteracting liver fibrosis genesis and portal hypertension progression with a concomitant ED improvement.
